# Development and Pharmacokinetic Evaluation of Novasomes for the Trans-nasal Delivery of Fluvoxamine Using Arachidonic Acid-Carboxymethyl Chitosan Conjugate

**DOI:** 10.3390/pharmaceutics15092259

**Published:** 2023-08-31

**Authors:** Saima Gulshan, Shahid Shah, Pervaiz Akhtar Shah, Muhammad Irfan, Malik Saadullah, Ghulam Abbas, Muhammad Hanif, Akhtar Rasul, Nabeel Ahmad, Abid Mahmood, Ejaz Basheer, Mohammad Omer Habib, Hadil Faris Alotaibi, Ahmad J. Obaidullah, Jawza F. Alsabhan, Osama l. Alwassil

**Affiliations:** 1 Department of Pharmaceutics, Faculty of Pharmaceutical Sciences, Government College University Faisalabad, Faisalabad 38000, Pakistan; saima.gulshan88@gmail.com (S.G.); manipharma@yahoo.co.uk (M.I.); akhtar.rasul@gcuf.edu.pk (A.R.); omergaba@gmail.com (M.O.H.); 2 Department of Pharmacy Practice, Faculty of Pharmaceutical Sciences, Government College University Faisalabad, Faisalabad 38000, Pakistan; shahid.waris555@gmail.com; 3 College of Pharmacy, University of the Punjab, Lahore 54000, Pakistan; pashah6512@yahoo.com; 4 Department of Pharmaceutical Chemistry, Faculty of Pharmaceutical Sciences, Government College University Faisalabad, Faisalabad 38000, Pakistan; maliksaadullah@gcuf.edu.pk (M.S.); abid9550@yahoo.com (A.M.); 5 Department of Pharmaceutics, Faculty of Pharmacy, Bahauddin Zakariya University Multan, Multan 60800, Pakistan; 6 School of Chemical and Materials Engineering, National University of Science and Technology, Islamabad 24090, Pakistan; nabeel.ahmed@scme.nust.edu.pk; 7 Department of Pharmacognosy, Faculty of Pharmaceutical Sciences, Government College University, Faisalabad 38000, Pakistan; eijaz.basheer@gmail.com; 8 Department of Pharmaceutical Sciences, College of Pharmacy, Princess Nourah Bint AbdulRahman University, Riyadh 11671, Saudi Arabia; hfalotaibi@pnu.edu.sa; 9 Department of Pharmaceutical Chemistry, College of Pharmacy, King Saud University, Riyadh 11451, Saudi Arabia; aobaidullah@ksu.edu.sa; 10 Department of Clinical Pharmacy, College of Pharmacy, King Saud University, Riyadh 11451, Saudi Arabia; jawza@ksu.edu.sa; 11 Department of Pharmaceutical Sciences, College of Pharmacy, King Saud bin Abdulaziz University for Health Sciences, Riyadh 11481, Saudi Arabia; wassilo@ksau-hs.edu.sa

**Keywords:** arachidonic acid-carboxymethyl chitosan (AA-CMCS) conjugate, fluvoxamine, antidepressant activity, mucoadhesion, permeation, pharmacokinetics

## Abstract

Depression is the major mental illness which causes along with loss of interest in daily life, a feeling of hopelessness, appetite or weight changes, anger and irritability. Due to the hepatic first-pass metabolism, the absolute bioavailability of fluvoxamine (FVM) after oral administration is about 50%. By avoiding the pre-systemic metabolism, nasal delivery would boost bioavailability of FVM. Additionally, the absorption is anticipated to occur more quickly than it would via the oral route because of the existence of microvilli and high vasculature. A nonionic surfactant, cholesterol and an arachidonic acid-carboxymethyl chitosan (AA-CMCS) conjugate were used to develop FVM-loaded novasomes. To investigate the effects of surfactant concentration, AA-CMCS conjugate concentration and stirring speed on the novasomes’ characteristics, a Box–Behnken design was used. The dependent variables chosen were zeta potential, polydispersity index and particle size. The AA-CMCS conjugate was confirmed by ^1^H-NMR and FTIR. Using Design Expert software (version 7; Stat-Ease Inc., Minneapolis, MN, USA), novasomes were further optimized. The chosen optimal formulation (NAC8) was made up of AA-CMCS conjugate, Span 60 and cholesterol. Particle size, zeta potential and PDI values for NAC8 formulation were 101 nm, −35 mV and 0.263, respectively. The NAC8 formulation’s DSC and TGA analysis demonstrated that the medication had been uniformly and amorphously distributed throughout the novasomes. The NAC8 formulation showed 99% and 90% FVM release and permeation, respectively, and the novasome adherence time was 24 h. An improved antidepressant effect along with five-fold increase in bioavailability of FVM was observed after trans-nasal administration of NAC8 formulation compared to the reference commercially available Flumin^®^ tablets. FVM-loaded novasomes administered via the nasal route may therefore constitute an advancement in the management of depression.

## 1. Introduction

Novasome as an innovative encapsulation process, originated by Novavax, IGI laboratories, is a technology designed to overcome various disadvantages associated with other drug delivery systems. As per their definition, they are a redefined form of liposomes or a variant of niosomes which contain a monoester of polyoxyethylene fatty acids, cholesterol and free fatty acids [1]. Along with several characteristics which include a multi-bilayer vesicle with an inner core with a large capacity in a specific size range, it also has the ability to deliver a significant amount of active ingredient. As a fatty acid-enriched vesicle, novasomes are believed to successfully penetrate the nasal membrane and increase the ability of the loaded medicine to enter the brain through the nose [2].

Fluvoxamine (FVM), 2-[(E)-[5-methoxy-1-[4 (trifluoromethyl) phenyl] petylidine] amino] oxyethaneamie, an antidepressant, is a core member of selective serotonin reuptake inhibitors (SSRIs), regarded as a second-generation antidepressant, controlled by the chemical class of 2-aminoethyl oxime ethers of aralkyl ketones. This substance is used by patients with obsessive compulsive disorder, anxiety disorder and depression. It reduces the symptoms of depression by increasing levels of extracellular serotonin in the brain blocking its uptake via the serotonin transporter. FVM blocks reutilization of the neurotransmitter named serotonin at the neuronal membrane, improving its behavior at receptors [3]. As a biopharmaceutical classification system (BCS) class II drug, FVM has low solubility and good penetrability. Data related to the absolute bioavailability of FVM when given orally show complete absorption and that food does not significantly interfere with its absorption [4]. Despite its complete absorption, the oral bioavailability of FVM falls in the range of 50–53% [4]. The main cause is first-pass metabolism which rendersits bioavailability to that lowest value. Oral formulations of FVM-like tablets and capsules pose many disadvantages regarding their onset of action and bioavailability which are both low and variable in the rate of absorption in different age groups. Oral treatment can be inconvenient because the slow onset of actions and low bioavailability of oral formulations can lead to long-term treatment which leads towards tolerance and withdrawal effects upon sudden termination of the therapy. The oral nanosuspension formulations of FVM which may also undergo first-pass metabolism are also reported in the literature [5,6,7]. Therefore, drug delivery through the nose provides fast action and a non-invasive therapy for depression. Due to its high permeability nasal delivery poses a great advantage which leads to higher bioavailability.

Carboxymethyl chitosan (CMCS), a linear polysaccharide, is a derivative of chitosan and cationic in nature. Due to this cationic nature, it forms electrostatic complexes and multilayer structures with other negatively charged synthetic or natural polymers. The most promising qualities of CMCS are that it is biocompatible, non-toxic, has a low tendency to cause allergies and is biodegradable [8]. For oral delivery, CMCS offers a major drawback due to its high viscosity, poor solubility under gastrointestinal physiological conditions which leads towards uncontrolled drug release. To take control of these drawbacks, the chemical modification of CMCS has been carried out by several researchers [9,10]. Mucoadhesion is the primary physiological component to be focused on for our trans-nasal delivery of FVM-loaded novasomes. For the novasomes to cross link with nasal mucus to lengthen adhesion duration and, eventually, improve bioavailability, CMCS specifically demonstrates the effective mucoadhesion property.

Hydrophobic linking of CMCS with a lipidic group can develop self-aggregate molecules in an aqueous medium. The lipophilic core of a lipid molecule offers good solubilization and high drug loading capacity for hydrophobic drugs. Lipids molecules, e.g., cholesterol, linoleic acid, stearic acid and arachidonic acid (AA), have been extensively used for the hydrophobic modification of chitosan derivatives [11]. Additionally, some reports showed that unsaturated fatty acids bearing a micellar system demonstrate a synergetic effect to an encapsulated active agent to improve the effectiveness of treatment. AA is a vital poly-unsaturated fatty acid, mainly used as surfactant-cleansing agent and emulsifying agent in cosmetic formulations. It is found in animal as well as human fat. As it is an integral part of cell membranes, that is why used as an absorption enhancer, as excipient is most of the formulations [12].

The goal of the current study is to develop the first trans-nasal FVM-based novasomes using an AA-CMCS conjugate. We hypothesized that AA-CMCS novasomes might enhance FVM solubility, prolong the drug release and improve mucoadhesion by taking advantage of their properties, such as improved solubility, small particle size, in vitro permeation and FVM’s antidepressant impact. Drug loading, in vitro release and physicochemical characterization (size analysis, zeta potential, FTIR, DSC and TGA) of the novasomes were adapted to select the most suitable formulation. Experimental rats were used to study the antidepressant and in vivo pharmacokinetics of the optimized NAC8 formulation.

## 2. Materials and Methods

### 2.1. Materials

Fluvoxamine (FVM) was gifted by Pharmevo Pharmaceuticals Pvt. Ltd, Lahore, Pakistan. Carboxymethyl chitosan (CMC) with Case number 83512-85-0 purchased from Glentham Life Sciences, Corsham, UK. CMC is white to light yellow or pale beige powder with 80% degree of deacetylation. Span 60, cholesterol, arachidonic acid (AA), methanol, 1-Ethyl-3-(3-dimethylaminopropyl)carbodiimide (EDC), N Hydroxysuccinimide (NHS), ethanol and potassium dihydrogen phosphate were purchased from Merck KGaA, Darmstadt, Germany.

### 2.2. Synthesis of Arachidonic Acid-Carboxymethyl Chitosan (AA-CMCS) Conjugate

**Figure 1:**
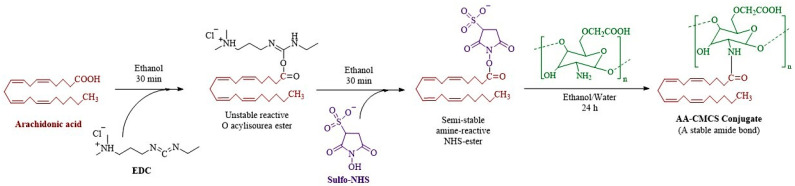
Chemical scheme for the formation of AA-CMCS conjugate.

For the preparation of the AA-CMCS conjugate, a reaction between the carboxyl methyl groups of the AA and the primary amino group of the low molecular weight CMCS was conducted. EDC/NHS was used as coupling agent. First, ethanol solutions of AA, EDS and NHS were prepared. The resulting solutions of AA, EDC and NHS were mixed, and the mixture was agitated for 30 min at 37 ± 5°C and thoroughly controlled for the activation of the carboxylic acid group of AA. Subsequently, a hydro-alcoholic (water: absolute ethanol: 9:1) solution of CMCS was prepared. Then, the ethanolic solution of AA and the CMCS solutions were combined by adding an ethanolic solution of AA drop wise in a hydro-alcoholic solution of CMCS. The resulting mixture was kept under stirring till overnight [13] and the resultant conjugate was characterized by ^1^H-NMR (Bruker Alpha, Karlsruhe, Germany) [14]. The chemical scheme for the formation of AA-CMCS conjugate is shown in Figure 1.

### 2.3. Experimental Design

Box–Behnken design was applied to design the formulation of FVM-loaded novasomes. Concentration of AA-CMCS conjugate, span 60 and stirring speed was selected as factors and size of particles, zeta potential, polydispersity index (PDI) and percent entrapment efficiency (%EE) as responses. A total of 17 formulations of novasomes were planned as shown in Table 1. The contour and 3D graphs were constructed and analyzed [15].

### 2.4. Preparation of FVM-Loaded Novasomes

Synthesis of novasomes that have been loaded with FVM was completed using an ethanol injection technique, using different ratios of span 60 and AA-CMCS conjugate. Initially, a mixture of span 60, AA-CMCS conjugate with 30 mg cholesterol and 50 mg FVM was prepared in 5 mL of ethanol. This solution was then placed in water bath for 1 h at a controlled temperature of 60 °C. This mixture was then injected slowly into 10 mL of phosphate-buffered saline solution (pH 7.4). The final product had a creamy texture after being mixed for two hours at a controlled temperature of 60 °C [16]. Drug loading is the mass ratio of drug to drug-loaded novasomes. The drug loading is calculated using Equation (1).

(1) $$Drug\;loading\;(\%)=\frac{Total\;amount\;of\;drug-Amount\;of\;drug\;in\;solution}{Total\;amount\;of\;formulation\;used}\times 100$$

### 2.5. %Entrapment Efficiency (%EE) of FVM

Separation of un-entrapped drug from 1 mL of freshly formulated novasome was conducted using a cooling preparative ultracentrifuge (Xiangzhi, Hunan, China) at the rate of 15,000 rpm for 1 h at a temperature of 4 °C. The supernatant (trapped) drug was then separated from residue (un-entrapped drug). After that, washing of the supernatant was conducted with ethanol for the removal of trapped drug from cavities between the novasomes and then washing of the supernatant was conducted again with ethanol for extensive removal of trapped drug from cavities between the novasomes, before it was re-centrifuged at 4 °C at a controlled rate of 15,000 rpm for 1 h. The supernatant was washed with the appropriate amount of distilled water to ensure complete removal of un-entrapped FVM. The absorbance of the supernatant was measured using a spectrophotometer (PerkinElmer, New York, NY, USA) after appropriate dilution at a wavelength of 272 nm taking ethanol as a blank. The FVM was quantified using an already developed calibration graph. Each test was performed three times [16].

(2) $$\%EE=\frac{Total\;amount\;of\;FVM-Un-entrapped\;FVM}{Total\;amount\;of\;FVM}\times 100$$

### 2.6. Analytical Characterization

The developed novasomes’ particle size was determined using a Malvern Zeta-sizer Nano (Nano Series ZS90) Malvern Panalytical Westborough, MA, USA, at an angle of 90° in 10 mm diameter cells at 25 °C. A total of 0.5 g of novasomes was diluted with 15 mL of distilled water for analysis. Mean effective diameter was used to describe particle size, while the polydispersity index was used to describe the width of the size distribution. A sample of the novasomes was diluted with demineralized water in a ratio of 1:40 to determine the zeta potential. All results were standardized to 20 °C as the reference temperature [17]. FVM, CMCS, AA, AA-CMCS conjugate, span 60, cholesterol, NAC8 blank and NAC8 FVM-loaded novasomes were characterized by FTIR (Bruker Alpha, Germany) with a wavelength range of 4000 to 400 cm^−1^ [17]. To evaluate the thermal stability of AA-CMCS, and the novasomes, thermogravimetric analysis (TGA) and diffraction scanning calorimetry (DSC) DSC-60 (Shimadzu, Düsseldorf, Germany) were used. To determine thermal stability, the weight loss against temperature increase was measured for the designed novasomes and AA-CMCS conjugate. The temperature was changed from 30 to 600 °C at a rate of 10 °C/min while dry nitrogen was applied continuously to 5 mg samples. There were three copies of each measurement.

### 2.7. In Vitro Release FVM from Novasomes

Using a USP dissolution device type II, research on the drug release from various FVM-loaded novasomes was carried out. FVM-loaded novasomes (equivalent to 2.5 mg FVM) were placed in a glass cylinder (3 cm in diameter and 8.5 cm in length) and the dialysis method was employed using a cellulose membrane. A cellulose sheath pre-soaked in simulated nasal fluid (prepared by dissolving 2.1925 g sodium chloride, 0.145 g calcium chloride and 0.745 g potassium chloride into 250 mL of double distilled water) was used to tightly cover each tube. The membrane was tied with a tube at one end and its other end was tied to the shaft of a peddle. Then, the shafts were dropped into the beakers, containing 50 mL of simulated nasal fluid as a dissolution vehicle, so that the outside of the cylinder touches the surface of the medium. To maintain a constant temperature throughout the experiment, beakers were jacketed inside the vessel of the apparatus. The adjustment of the cylinder was made so that it rotated at a continuous speed of 100 rpm at 37 °C. Throughout the experiment, the vessels were covered permanently for the purpose of lessening the evaporation of medium. The samples were taken at continual determined time intervals (0.5, 1, 2, 3, 4, 5, 6, 7 and 8 h), each sample contained a volume of 3 mL and after drawing each sample, it was substituted with fresh medium to maintain sink conditions and measurements were taken spectrophotometrically at a wavelength of 272 nm while using simulated nasal fluid as a blank. The results were taken as mean values (*n* = 3 ± SD) [16].

### 2.8. FVM Release Kinetics

For the assessment of the percentage release kinetics of FVM, different kinetics models were applied such as Zero order, First order, Higuchi model, Hixson–Crowell and Korsmeyer–Peppas models [18].

### 2.9. Permeation Studies

The permeation study of FVM-loaded novasomes was evaluated the using freshly cut nasal mucosa of sheep. The NAC8, Flumin tablet and the FVM were applied to the mucosal surface of the nasal cavity fixed to the bottom of the beaker. The beaker contained 100 mL of bicarbonate Ringer solution maintained at pH 7.4 and stirred at a rate of 150 rpm at 37 °C. The permeation time was the time needed for complete permeation of the drug from the mucosal surface. Absorbance data was calculated at 272 nm via a UV-visible spectrophotometer [19].

### 2.10. Mucoadhesion Studies

The mucoadhesion of novasomes was performed on the freshly excised nasal mucosa of sheep. A glass slide was taken, adhered the nasal mucosa using cyanoacrylate glue and a sheet of sheep nasal mucosa was fitted on it. The CMCS, AA-CMCS conjugate and novasomes (10 mg each) were attached to the nasal mucosal membrane by finger press. A modified USP disintegration apparatus type II containing simulated nasal fluid pH 7.4 at 37 °C was used at 50 rpm. The glass slide fixed with nasal mucosa and preparations were attached with a peddle of dissolution apparatus and the time was noted when applied preparations were detached from the nasal mucosa [20].

### 2.11. Cell Viability Assay

To study the cell toxicity of FVM, CMCS, AA-CMCS conjugate, AA, NAC8 blank and NAC8 FVM-loaded novasomes, An MTT assay was performed on the MCF-7 cell line (obtained from the American Type Culture Collection (ATCC, Manassas, VA, USA). In culture media (Dulbecco’s Modified Eagle Medium with 10% fetal bovine serum), the cells with passage number 20 were cultured in 96-well plates (FBS). After that, cells were incubated for 6 and 24 h in FBS-free growth media containing 0.5% dispersions of various samples. The cells were rinsed with phosphate-buffered saline following incubation (1X PBS). Following the addition of 500 µL of MTT solutions in FBS-free media (0.5 mg/mL), the cells were once more incubated for 1 h. After draining the supernatant, 500 µL of DMSO was added to each well to solubilize the transformed dye. The absorbance at 570 nm was then measured. The percentage of cell viability was then determined using the following equation: [21].

(3) $$Cell\;viability\;\left(\%\right)=\frac{A_{s}}{A_{d}}\times 100$$

In Equation (2) *A_s_* and *A_d_* are absorbances of sample dispersions and DMEM.

### 2.12. Anti-Depressant Activity

#### 2.12.1. Animals

An experiment on male Sprague-Dawley rats (25–30 g) 61–65 days old was conducted for the evaluation of antidepressant activity and in vivo behavioral studies. Before conducting any experiments, the animals were kept in a cycle of light and dark under ambient conditions at 20 °C with a 12 h period for each cycle and they were given free access to a standard diet and tap water for 3 days. International Council for Harmonisation (ICH) guidelines were followed during the performance of experiments on animals. Three groups were made, and each group contained3 rats [18]. At the end of the experiments all animals were sacrificed using cervical dislocation under anesthesia (2–4% isoflurane inhalant).

#### 2.12.2. Preparation of Test Samples

Each medicament was administered at the dosage of 1 mg/kg. Rats in the control group were given 0.5% aqueous carboxymethyl cellulose (CMC) suspension used as vehicle. Test drug FVM-loaded novasomes was administered using a trans-nasal drug delivery system through nose. The FVM-loaded novasomes in purified water were dispersed and with the help of micropipette administered into the nostrils of the rats. FVM in 0.5% CMC was used as reference medication and administered orally via feeding tube [18,22]. The respective groups received experimental drugs for a total of 28 days. The rats were prepared for a forced swimming and tail suspension test on day 28.

#### 2.12.3. Forced Swimming Test

The anti-depressant activity of developed novasomes was checked by the forced swimming test (FST) described by Butterweck et al., 2001 with minor modifications [23]. The test sample was administered through nasal application (test group) while oral administration of a reference medication (reference group) was performed. The control group received only 1 mL of deionized water through nasal application. One hour after administration of doses, animals were placed individually in 20 cm high glass beaker which was transparent and filled with tap water at 23 ± 2 °C, water was filled to a height of 10 cm and rats were forced to swim. Water was replaced with fresh water after each experiment and each rat was used only one time for one experiment. Video recording of all the experiments was carried out and the total duration of activity was chronometered for the last 2 min of a 6 min long duration period. Animals were considered immobile when they did make any effort to escape from the glass beaker, except for the activities needed to hold their hands in water [18,24]. This test is used to monitor depressive-like behavior and is based on the assumption that immobility reflects a measure of behavioral despair.

#### 2.12.4. Tail Suspension Test

The test sample was administered through nasal application (test group) while oral administration of reference medication (reference group) was performed. The control group received only 1 mL of deionized water through nasal application. One hour after administration of doses, the rats were suspended individually 50 cm above the floor at 1 cm from the tip of the tail through an adhesive tape. Video recording of all the experiments was carried out and total duration of inactivity was scored for the last 6 min of 10 min long session. Immobility was measured when the rats did not make any attempt to get rid of the tape for at least 1 min [18,25].

### 2.13. HPLC Method for FVM Analysis

We developed a high performance liquid chromatography (HPLC) method for the quantification of FVM from the plasma samples. As an internal standard, clovoxamine was used in the procedure. A heptane-isopropanol mixture was used to extract the serum in alkaline medium (NaOH 2M), which was then followed by an extraction in hydrochloric acid (0.1N). The aqueous phase was then injected in a volume of 30 µL onto a Nucleosil C8 µ 5 column (150 × 4.6 mm). The mobile phase has a flow rate of 1.2 mL/min and was composed of an acetonitrile and phosphate-buffered solution with a pH of 7.4 (50:50 *v*/*v*). The chosen absorption wavelength was 190. Internal standard retention time was 4.9 min, while FVM retention duration was 6.5 min.

### 2.14. Pharmacokinetics of FVM

For the pharmacokinetic experiments, three groups of six albino rats each weighing between 250 and 450 g was chosen: a test group, reference group and a control group. The albino rats were 9–11 weeks old were obtained from the animal house of the Department of Pharmaceutics Government college, University Faisalabad. The rats were kept at 25 °C with relative humidity of 45%. Government College University Faisalabad, Punjab, Pakistan’s Ethical Committee for the Utilization of Laboratory Animals approved the pharmacokinetics evaluation with approval number 0098, 12 February 2022. When conducting a pharmacokinetic study, albino rats were handled in accordance with the ICH (International Council for Harmonization) guidelines. Over the course of time, the temperature stayed at 25 °C. Prior to starting the pharmacokinetic investigation, the albino rats were fasted for 24 h while still being given free access to water. Using a micropipette, suspension of FVM and novasomes of NAC8 containing (1 mg/kg body weight of albino rats) FVM were instilled into each nostril of the rats of the control and test groups. The albino rats were supported from behind and tilted during trans-nasal administration. The formulations were instilled into the nasal apertures. The treatment was carried out delicately so that the animals could breathe through all the preparation. The reference formulation Flumin^®^ tablet was administered orally using a feeding tube to the reference group. Following administration, blood was taken into ethylene diaminetetraacetic acid tubes at 1, 2, 4, 6, 8, 12, 24, 36 and 48 h. Centrifugation at 4500 rpm was used to instantly separate the plasma samples. Before being analyzed, plasma samples were kept at −20 °C. The pharmacokinetics parameters such as t_max_, C_max_, AUC, t_1/2_, AUC and MRT were calculated.

### 2.15. Statistical Analysis

The Statistical Package for Social Sciences (SPSS, version 25.0) was used for data analysis. The statistical difference of different treatments was analyzed through analysis of variance. It included the evaluation and calculation of means, standard deviation and group comparisons using Dunnett’s test. The confidence interval was set up to 95% and *p* < 0.05 was considered statistically different [16].

## 3. Results and Discussion

Antidepressants must typically be delivered to the brain region affected by early and advanced stages of depression in order to achieve the desired therapeutic effect. Drug delivery via the nasal route promotes enhanced absorption and prevents the first-pass impact. Mucoadhesive drug delivery systems may be able to resist the wash-out of drug formulation from the nasal cavity because nasal fluid continuously irrigates the nasal canal and provides protection from particle and microbial invasion. Additionally, mucoadhesive drug delivery systems promote targeted medication delivery to specific brain regions and facilitate drug absorption. In contrast to nanoparticles, novasomes have semisolid cream-like dose forms that can be administered to the mucosal surface with ease.

### 3.1. Synthesis and Characterization of AA-CMCS Conjugate

After the reaction, analysis through ^1^H-NMR of the reaction material was conducted, and results showed that the AA-CMCS conjugate was successfully prepared [26]. Amide bond formation between the carboxyl group of AA and the amine bond of CMCS was responsible for the formation of AA-CMCS conjugate. The CMCS ^1^H-NMR spectrum exhibits signals in the range of 4.14–4.60 ppm that correspond to two hydrogens of the carboxymethyl group linked to C_6_ and one hydrogen of the carboxymethyl group bonded to C_3_ as shown in Figure S1. The signals in the range of 3.55–3.95 ppm were allocated to the two hydrogens from the carboxymethyl group bound to the nitrogen group [27]. In AA, the olefinic protons of conjugated double bonds and glycerol moiety are in the range of 5.52–5.35 ppm and 3.64–2.68 ppm, respectively, as shown in Figure S2. The -CH_2_ and CH_2_-CH=CH protons are in the range of 2.26–2.10 ppm and 2.04 ppm, respectively. The (CH_2_)n and CH_3_ protons in the regions of 1.65–1.29 ppm and 0.93–0.82 ppm, respectively [28]. The appearance of a characteristic ring of methine protons of AA-CMCS conjugate spectra in the region of 5.55–5.44 ppm proved the grafting of CMCS with AA in the AA-CMCS conjugate as shown in Figure S3. Also, the broadening of peaks of (CH_2_)n and conjugated double bonds were observed at 1.7 ppm and 5.5 ppm in the spectra of the AA-CMS conjugate which was due to the presence overlap of the methylene proton adjacent to a double bond with the acetamide group protons [26].

### 3.2. Preparation of Novasomes and Experimental Design

To prepare novasomes with the desired characteristics, 17 formulations were prepared with different types and ratios of surface-active agents (span 60) and free fatty acids (FFAs) conjugate with AA while keeping the concentration of cholesterol same [29]. The Box–Behnken design was effectively utilized to evaluate the effects of factors on the particle size of novasomes, zeta potential, PDI and %EE. The concentration and value of factors and the observations of responses are listed in Table 1. The contour and 3D graphs were generated as shown in Figure S4, to study the effect of the AA-CMCS conjugate, stirring speed and surfactant on size of particles, zeta potential and PDI of the developed novasomes. As the stirring speed increased, the particle size decreased. The value of *p* for particle size was 0.0004, obtained from ANOVA results indicating the significance of the results. In lack of fit, the value of *p* was 0.9696 indicating the non-significance of the results. The value of the zeta potential moved towards a more negative value when the concentration of modified chitosan was 1.5 g compared to 1 and 2 g formulations. The value of *p* for the zeta potential was 0.0006, obtained from ANOVA results indicating the significance of the results. In lack of fit, the value of *p* was 0.6356 indicating the non-significance of results. In PDI, the value was lowest when the concentration of modified polymer was 1 g as compared to the formulation containing1.5 and 2 g. The value of *p* for PDI was 0.0186, obtained from ANOVA results indicating the significance of the results. In lack of fit, the value of *p* was 0.034. The value of *p* for %EE was 0.765, obtained from ANOVA indicating the results were statistically insignificant. In lack of fit, the value of *p* for %EE was 0.6356 indicating the non-significance of the results.

### 3.3. % Entrapment Efficiency (%EE)

The %EE is an important parameter in the design of novasomal formulations. The %EE of novasomes depends upon the stability of novasomes which depends upon the type of surfactant [30]. The values of %EE and drug loading are mentioned in Table S1. The results indicated that the FVM was successfully loaded in the novasomal formulations and the highest %EE was achieved for the NAC8 formulation.

### 3.4. Particle Size and Zeta Potential Analysis

**Figure 2:**
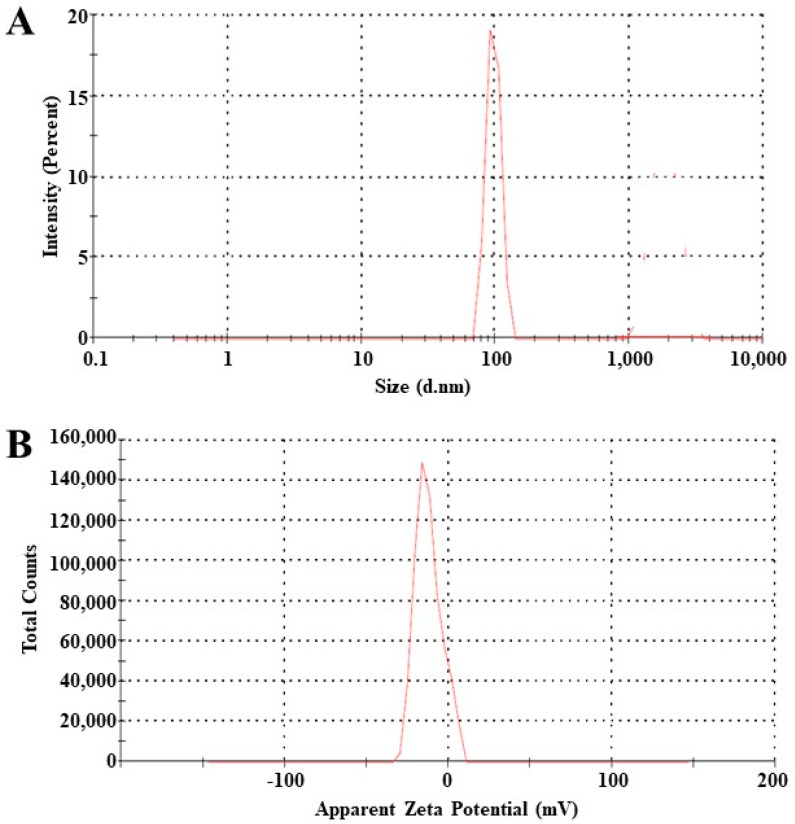
Particle size (**A**) and zeta potential (**B**) of NAC8 novasomes formulation (*n* = 6).

Particle size, zeta potential and PDI play crucial roles in regulating a variety of processes, including medication absorption, release rate and stability. There have been reports of drug bioavailability and absorption increase at diameters less than 300 nm [31]. The average diameter of an NAC8 globule was measured as 101 nm as shown in Figure 2A. The PDI of the formulation was 0.263 which was less than 0.4. The stability of the novasomes depends on the zeta potential as it opposes aggregation [32]. The zeta potential of all prepared novasome formulations ranged from −12 to −35, out of these NAC8 was the most stable as shown in Figure 2B.

### 3.5. Fourier Transforms Infrared Spectroscopy (FTIR)

**Figure 3:**
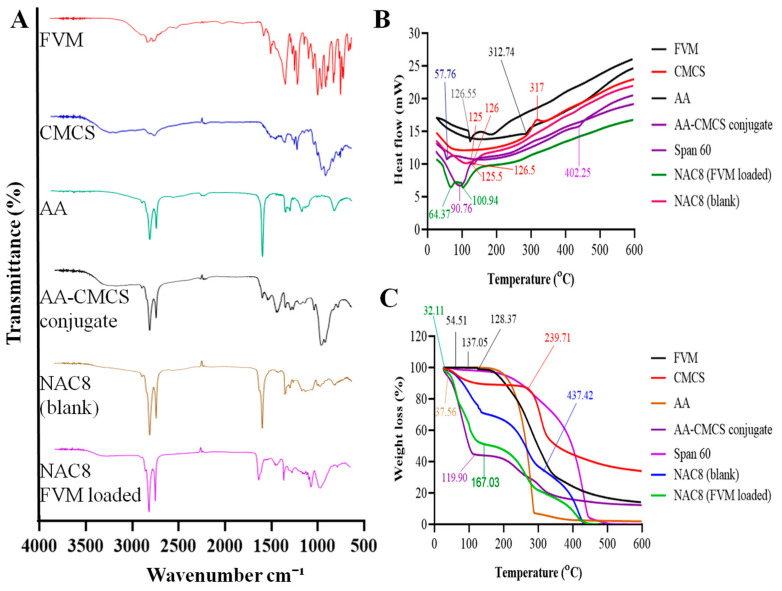
FTIR (**A**), DSC (**B**) and TGA (**C**) of FVM, CMCS, AA, AA-CMCS conjugate, NAC8 blank and NAC8 FVM-loaded novasomes.

FVM showed a stretching of the C-H group at 2995 cm^−1^ as shown in Figure 3A. The peaks of the acidic O-H group were observed at 2880 cm^−1^. A weak peak of -C=O stretching of the acidic group was observed at 1703 cm^−1^ and N-H bending of the secondary amine group was observed at 1621 cm^−1^. Two peaks were observed at 1493 cm^−1^ and 1362 cm^−1^ due to C-H bending and C-F stretching of ether [33]. Strong peaks were observed between 862 cm^−1^ and 989 cm^−1^ due to the para-substituted aryl group. The spectra of CMCS showed stretching of the amine group, absorption of the -OH group, C=O stretching and bending of the C-H group at 3413 cm^−1^, 2890 cm^−1^, 1613 cm^−1^ and 1440 cm^−1^, respectively [34]. In the spectra of AA, the stretching vibrations of the C=C and C=O of carboxyl groups and -CH2 were observed at 1492 cm^−1^, 1705 cm^−1^ and 2835 cm^−1^, respectively [35]. The presence of a peak at 1640 cm^−1^ in the AA-CMCS conjugate indicated the formation of an amide bond between CMCS and AA. Blank showed peaks at 2890 cm^−1^, 2835 cm^−1^, 1640 cm^−1^ and 1440 cm^−1^, which corresponds to the peaks of CMCS, AA and the AA-CMCS conjugate. The drug-loaded formulation showed peaks at 2895 cm^−1^, 2839 cm^−1^, 1900 cm^−1^ and 1550 cm^−1^ which indicated the peaks of the AA-CMCS conjugate and span 60, and the peaks of FVM disappeared indicating the uniform distribution of FVM in the novasomes.

### 3.6. Differential Scanning Calorimetry (DSC)

DSC is one of the methods often employed to examine the physical and chemical interaction between an excipient and a medicinal substance. FVM showed an endothermic peak at 125.55 °C [35] which represented the melting point of drug. CMCS showed exothermic peak at 317 °C [36] which corresponds to the degradation of the polymer and AA showed an endothermic peak at 312.74 °C. The AA-CMCS conjugate showed an endothermic peak at 90.76 °C [37]. Span 60 showed two endothermic peaks at 57.76 °C and 402.25 °C [38]. The drug-unloaded sample showed one exothermic peak at approximately 125 °C followed by an endothermic peak at 125.5 °C and an exothermic peak at 126.5 °C. The drug-loaded sample showed two endothermic peaks at 98.06 °C and 100.94 °C as shown in Figure 3B. Due to the presence of FVM in amorphous rather than crystalline form, the endothermic peak of FVM in optimized NAC8 novasomes was somewhat different from that of pure FVM due to the proper entrapment of the drug in formulation. DSC testing demonstrated compatibility between the FVM and the AA-CMCS conjugate.

### 3.7. Thermogravimetric Analysis (TGA)

TGA is typically used to examine a material’s thermal performance. The TGA of FVM indicated 0.94% weight loss up to 128.37 °C related to the removal of absorbed water from the FVM as shown in Figure 3C [39]. The CMCS showed a weight loss of 1.07% at 35.99 °C, while 11.42% at 239.71 °C [40]. The mass loss below 100 °C in the CMCS may be attributable to the evaporation and volatilization of water. However, it was observed that the primary skeleton of the CMCS started to decompose at about 110 °C. At a temperature of about 599 °C, 65% of the CMCS thermally decomposed. AA showed a weight loss of 0.10% at 137.05%, while 93.08% at 295.15 °C [41]. The AA-CMCS conjugate showed a weight loss of 55.54% at 119.90 °C, while Span 60 had a weight loss of 0.50% at 54.51 °C. The TGA curve of NAC8 novasomes presented two mass loss stages. The first stage was due to the evaporation of adsorbed and bound water in the temperature range of 30–167.03 °C. In the second stage, the maximum degradation rate of the novasomes occurred between 168 and 431 °C due to the thermal depolymerization of the polymeric conjugate.

### 3.8. Release of FVM from Novasomes

**Figure 4:**
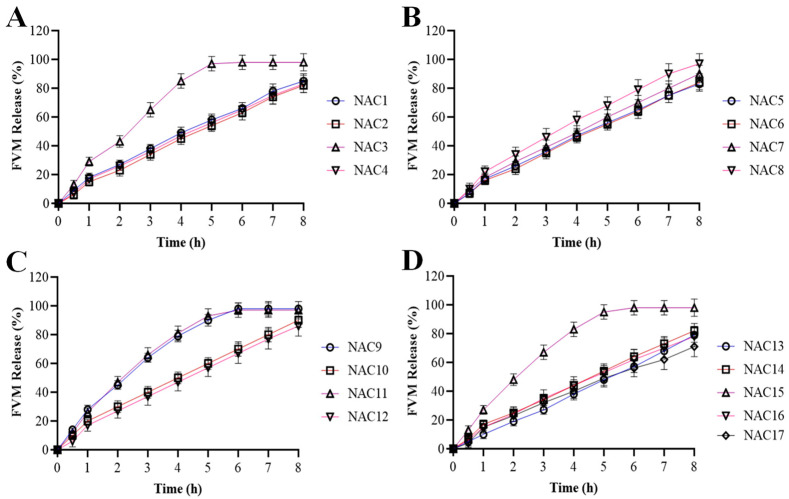
Release profile of FVM from novasome formulations containing different ratios of AA-CMCS conjugate and span 60; NAC1 to NAC4 (**A**), NAC5 to NAC8 (**B**), NAC9 to NAC12 (**C**) and NAC13 to NAC17 (**D**) in phosphate-buffered saline ph 7.4 for up to 8 h.

Drug release studies of our formulations from NAC1 to NAC17 were performed [42]. These novasomes were prepared by changing the ratios of Span 60 and AA-CMCS conjugate. Formulation of the NAC1 to NAC4 release pattern is shown in Figure 4A. It indicates that NAC3 showed a rapid increase following a steady release of 85% at 4 h with particle size 289 nm, while NAC1, NAC2 and NAC4 showed uniform release up to 80% at 8 h. The particle size of the NAC4 was 108 nm which displayed the more sustained release pattern compared to the NAC1. Figure 4B shows the percent release of NAC5 to NAC8. NAC5 to NAC7 showed a uniform release of the drug up to 85% while NAC8 showed a maximum release up to 99% at 8 h, which is also regarded as our best formulation having an AA-CMCS to Span 60 ratio of 1.5:1. The particle size of NAC8 was 101 nm and indicated that the smaller particle size of novasomes showed a more sustained effect compared to the formulations with a greater particle size. The release pattern of the novasomes formulation (NAC8) showed that the drug was released within 8 h. NAC9 to NAC12 showed a maximum release of up to 80% in which NAC9 and NAC11 showed a release of up to 85% then a constant release pattern as shown in Figure 4C. NAC13 to NAC17 followed the same pattern as NAC1 to NAC4 with a release percent of up to 80% at 8 h as shown in Figure 4D. From the following data it can be interpreted that by increasing the AA-CMCS concentration there was an increase in the release pattern on FVM-loaded novasome while span 60 also contributed to this factor to an extent. The formulation NAC8 with a high AA-CMCS conjugate was labelled as our best formulation having up to a 99% release with low particle size and zeta potential values, and a PDI less than 0.3.

### 3.9. Release Kinetics

For the evaluation of in vitro release studies of FVM-loaded novasomes different kinetic models were applied. The value of R² in zero order models ranged from 0.8151 to 0.9987, while the value of R² in first order models ranged from 0.7571 to 0.8926. In the Higuchi model values R^2^ varied from 0.8599 to 0.9635 as shown in Table S2. Values of R^2^ in the Hixson–Crowell model were from 0.9752 to 0.9963. In the Korsmeyer–Peppas model values of n ranged from 0.413 to 0.490 which is, as can be seen, less than ˂0.5 which shows that our drug followed Fickian diffusion model [43]. To understand the mode and mechanism of drug release, the in vitro FVM data were converted and evaluated using various kinetic models. Several kinetic models were fitted using the FVM in vitro release data. The findings are shown in Table S2. By entering the release data into the Korsmeyer–Peppas equation, the mechanism of the drug release can be identified according to the value of the release exponent “n.” It is obvious that the novasomes underwent Fickian diffusion because the values of the release exponent “n” ranged from 0 to 0.5.

### 3.10. Permeation Studies

**Figure 5:**
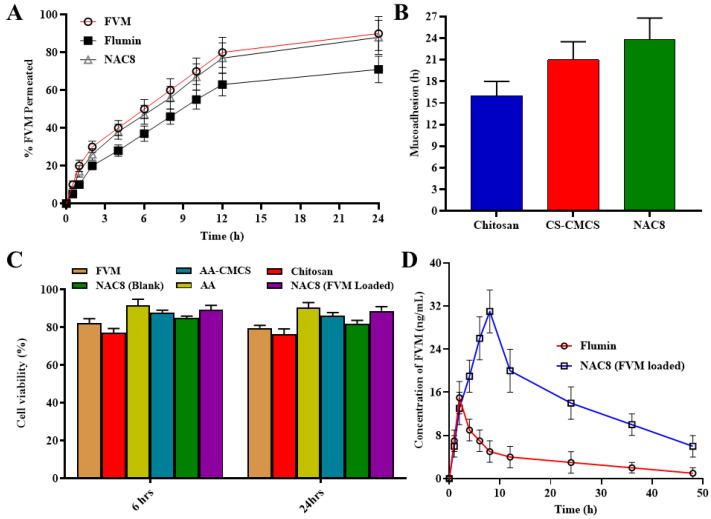
Permeation (**A**), mucoadhesion (**B**) cell viability study (**C**) of FVM, AA-CMCS conjugate, blank and FVM-loaded novasomes and (**D**) peak concentration of FVM from Flumin reference formulation after oral administration and NAC8 (FVM-loaded) test formulation after trans-nasal administration (*n* = 6).

Permeation studies were conducted up to a 24 h period. And permeation results were interpreted for our drug FVM, the marketed product Flumin and the NAC8 formulation which was identified as our best formulation. FVM alone showed a % permeation up to 90% in a 12 h period while the % permeation of Flumin was below 80% as shown in Figure 5A. The NAC8 formulation showing the permeation of FVM from novasome was above 85%. As per the given results, it is concluded that the modification of CMCS in the AA-CMCS conjugate led towards increased mucoadhesion of our formulation to nasal mucosa. So, in turn, increasing mucoadhesion was followed with enhanced absorption to increase the percent release [20].

### 3.11. Mucoadhesion Study

The mucoadhesion study of chitosan, AA-CMCS conjugate and the FVM-loaded novasomes NAC8 formulation have been shown in Figure 5B with a significant difference of their time and duration of adhesion to mucosa, which have been studied up to a period of 24 h. The CMCS remained adhered to mucosa for up to 16 h, while the AA-CMCS had an adherence time of about 21 h andNAC8 formulation had an adherence time of up to 24 h. The adherence of AA-CMCS was double that of CMCS alone as the AA-CMS conjugate had amide linkage which led to its enhanced mucoadhesion. The NAC8 formulation with the AA-CMCS conjugate showed mucoadhesion up to a 24 h period which was our desired characteristic when formulating the modified chitosan. An increase in mucoadhesion was our desired parameter in terms of our formulation so that is the drug remains adhered to the nasal mucosa for a maximum length of time leading to a constant release of the drug [20].

### 3.12. Cell Viability

As a result of the cell viability studies conducted it was observed that the viability of cells remained up to 90% which shows that our formulation is non-toxic and safe. The viability of MCF-7 cells treated with drug-loaded novasomes was more than 80% in comparison to the viability of the cells handled with the drug-unloaded formulation. Cells that were treated with the formulation demonstrated viability ranging from 85% to 90% after 6 h and about 85% after 24 h. The variation between 6 and 24 h was not significant as shown in Figure 5C. The results indicate that novasome dosage forms have a slightly different toxicity profile and safety. However, it can be assumed that the developed novasomes are safe to use since they do not significantly affect the viability of cells [44].

### 3.13. Antidepressant Activity

The results of the forced swimming test and tail suspension tests are summarized in Table 2. The rat’s immobility displays human-like behavioral despondency. Antidepressants are basically used to treat the symptoms and reduce the duration of inactivity to any response. If the subject showed any locomotive activity in any stressful situations with a shortened duration of inactivity and a response to any trigger it was considered a positive response of the rats. The results showed a significant decrease in the duration of immobility of rats with FVM-loaded novasomes compared to the control and pure drug. The decrease in duration of inactivity shows a somewhat rapid response of rats towards a stressful situation which in turns explains the rapid onset of action as compared to the pure drug. As FVM-loaded novasomes were administered through nasal delivery they lead towards rapid absorption and quick onset of action compared to the pure drug which was given orally [45].

### 3.14. Pharmacokinetics of FVM from Novasomes

Over the course of 48 h, the plasma concentration of FVM was assessed via oral and trans-nasal routes. The observed plasma concentration and time were plotted on a graph (Figure 5D). It took an average of 2.23 ± 0.70 h for oral administration and 8.28 ± 1.56 h for trans-nasal administration to reach the maximum plasma concentration (t_max_), respectively, (*p* = 0.019) as shown in Table 3. In contrast to the oral route (11.46 ± 2.09 h), the trans-nasal route had a longer t_1/2_ (25.63 ± 3.21 h). Although the difference in our study group was statistically significant (*p* = 0.015), we saw a similar pattern. It was observed that the mean FVM plasma levels observed after oral administration (AUC = 113.52 ng × h/mL, C_max_ = 11.46 ng/mL) are less to those obtained by trans-nasal administration in albino rats (AUC = 707.26 ng × h/mL; Cmax = 25.63 ng/mL) [46]. The oral bioavailability was enhanced five-fold after trans-nasal administration. Thus, the difference of pharmacokinetic parameters between oral and trans-nasal routes may be due to the first-pass effect occurring after oral administration of FVM. Thus, after trans-nasal administration, the C_max_ was about doubled and the AUC was almost six times higher. The AUMC of FVM after trans-nasal administration was 1.2 times higher compared to the oral route.

The Pharmacokinetics of FVM from NAC8 novasomes was also compared with the control group (received FVM suspension). A one-fold and three-fold increase in the C_max_ and T_max_ of NAC8 novasomes was observed compared to control group as mentioned in Table 3. The half-life (t_1/2_), AUC and MRT of NAC8 was increased one-fold compared to the control group. There was a 2.3 times higher bioavailability of FVM from the NAC8 formulation compared to the control group.

## 4. Conclusions

Novasomes of the AA-CMCS conjugate containing FVM were developed and tested in an effort to create an efficient and long-lasting trans-nasal nano-based system for the treatment of depression. Following the application of various formulations and the analysis of optimization data, the formulation (NAC8) was chosen for further evaluation. Significant physicochemical characteristics of NAC8 included a wide variety of nanometric sizes, a moderate zeta potential and high entrapment effectiveness. Studies using FTIR and DSC demonstrated that FVM and additives are compatible. Studies on the in vitro release of novasomes revealed a longer and better release profile, with 23.5 ± 1.49% and 99.5 ± 1.92% released after 2 h and 8 h, respectively. In vivo experiments indicated that the developed novasomes were more effective at treating depression than commercial Flumin^®^, and they also confirmed the promising potential of FVM in the treatment of depression. A five-fold increase in bioavailability of FVM was observed after trans-nasal administration of the NAC8 formulation. The pharmacokinetics of the NAC8 formulation showed a bioavailability 2.3 times higher than that of the control group (FVM suspension) administered nasally. The mucoadhesion, permeation and bioavailability of the NAC8 novasome formulation were remarkably higher than those of the control group.

## Figures and Tables

**Table 1:** Factors and responses for Box–Behnken design.

Run	AA-CMCS Conjugate (mg)	Span 60 (mg)	Stirring Speed (rpm)	Particle Size (nm)	Zeta Potential (mV)	PDI	%EE
NAC1	1.5	2.0	500	340 ± 5.9	−12 ± 2.1	0.543 ± 0.002	59.56 ± 1.453
NAC2	1.5	1.5	750	278 ± 5.1	−19 ± 2.5	0.342 ± 0.004	67.34 ± 1.089
NAC3	1.0	2.0	750	289 ± 4.2	−17 ± 3.1	0.378 ± 0.005	71.67 ± 2.981
NAC4	1.5	2.0	1000	108 ± 4.1	−27 ± 2.1	0.305 ± 0.008	56.45 ± 1.652
NAC5	1.5	1.5	750	256 ± 3.9	−21 ± 1.6	0.299 ± 0.006	78.40 ± 1.789
NAC6	2.0	1.5	1000	102 ± 3.6	−28 ± 1.5	0.213 ± 0.014	81.34 ± 1.021
NAC7	1.5	1.0	500	356 ± 6.1	−13 ± 1.9	0.421 ± 0.005	49.81 ± 2.098
NAC8	1.5	1.0	1000	101 ± 3.1	−35 ± 2.6	0.263 ± 0.001	90.92 ± 1.567
NAC9	1.0	1.0	750	226 ± 4.8	−20 ± 3.2	0.341 ± 0.013	82.45 ± 1.043
NAC10	1.5	1.5	750	209 ± 3.8	−19 ± 2.7	0.305 ± 0.005	80.90 ± 1.498
NAC11	1.0	1.5	1000	106 ± 5.2	−33 ± 2.0	0.293 ± 0.004	67.92 ± 1.619
NAC12	1.5	1.5	750	291 ± 3.8	−15 ± 2.1	0.376 ± 0.008	75.39 ± 2.923
NAC13	2.0	2.0	750	243 ± 4.6	−17 ± 3.1	0.313 ± 0.003	63.34 ± 3.678
NAC14	2.0	1.5	500	339 ± 4.6	−16 ± 2.5	0.409 ± 0.015	53.21 ± 2.937
NAC15	1.0	1.5	500	348 ± 5.3	−15 ± 2.4	0.412 ± 0.006	59.82 ± 3.452
NAC16	1.5	1.5	750	227 ± 4.6	−20 ± 1.5	0.301 ± 0.004	78.45 ± 3.765
NAC17	2.0	1.0	750	271 ± 4.1	−21 ± 2.3	0.410 ± 0.007	80.67 ± 3.591

**Table 2:** Values of immobility for the forced swimming and tail suspension tests.

Material	Dose	Duration of Immobility (Seconds)
Forced swimming test		
Control	1 mL/kg	176 ± 2.88
Pure Drug (FVM)	1 mg/kg	151 ± 3.89
FVM-loaded novasomes (NAC8)	1 mg/kg	120 ± 5.64
Tail suspension test		
Control	1 mL/kg	126 ± 5.44
Pure Drug (FVM)	1 mg/kg	109 ± 4.65
FVM-loaded novasomes (NAC8)	1 mg/kg	86 ± 3.23

**Table 3:** Parameters of pharmacokinetics of FVM from Flumin^®^ (reference formulation), FVM suspension (control group) and NAC8 FVM-loaded (test formulation) novasomes.

Parameters	Units	FVM Suspension	Flumin^®^	NAC8 FVM-Loaded Novasomes
C_max_	ng/mL	12.75 ± 2.78	11.46 ± 2.09	25.63 ± 3.21
T_max_	h	2.01 ± 0.93	2.23 ± 0.70	8.28 ± 1.56
t_1/2_	h	7.72 ± 1.34	5.08 ± 1.20	15.39 ± 2.02
AUC_0−t_	ng × h/mL	349.31 ± 4.21	113.52 ± 4.23	707.26 ± 5.43
AUC_0−ꝏ_	ng × h/mL	415.39 ± 3.49	113.71 ± 2.10	821.66 ± 6.32
AUMC	ng × h/mL	1053.49 ± 6.09	940.99 ± 6.91	21,402.99 ± 8.21
MRT	h	12.87 ± 1.23	8.27 ± 1.09	26.048 ± 2.04

## Data Availability

The data presented in this study are available in this article (and Supplementary Materials).

## Supplementary Materials

The following supporting information can be downloaded at: https://www.mdpi.com/article/10.3390/pharmaceutics15092259/s1. Figure S1. 1H-NMR of CMCS. Figure S2. 1H-NMR of AA. Figure S3. 1H-NMR of AA-CMCS. Figure S4. Contour and 3D graphs showing the effect of AA-CMCS conjugate, span 60 and stirring speed on the particle size (A and B), zeta potential (C and D) and PDI (E and F). Table S1. Values of %EE and % drug loaded in novasomes. Table S2. The kinetic parameters of FVM release from novasomes at pH 7.4.
