# Supplementary material for: Development and Pharmacokinetic Evaluation of Novasomes for the Trans-nasal Delivery of Fluvoxamine Using Arachidonic Acid-Carboxymethyl Chitosan Conjugate
Source: Pharmaceutics. 2023 Aug 31;15(9):2259. doi: 10.3390/pharmaceutics15092259 (PMC10535484; doi:10.3390/pharmaceutics15092259)
Supplement: Supplementary file 1 [file pharmaceutics-15-02259-s001.zip › pharmaceutics-2487892-supplementary.pdf]

Novasomes for the transnasal delivery of fluvoxamine using arachidonic acid-carboxymethyl chitosan conjugate: Permeation, mucoadhesion and pharmacokinetic evaluation

1. (Proton-nuclear magnetic resonance) ^{1}H-NMR spectroscopy

^{1}H-NMR of carboxymethyl chitosan (CMCS)

^{1}H NMR, δ 4.66 (dq, 1H), 4.23 (d, 1H), 4.14 (s, 2H), 3.92 (q, 1H), 3.90 – 3.83 (m, 2H), 3.83 – 3.71 (m, 2H), 3.69 (dd, 1H), 3.65 – 3.55 (m, 2H), 3.42 – 3.35 (m, 8H)

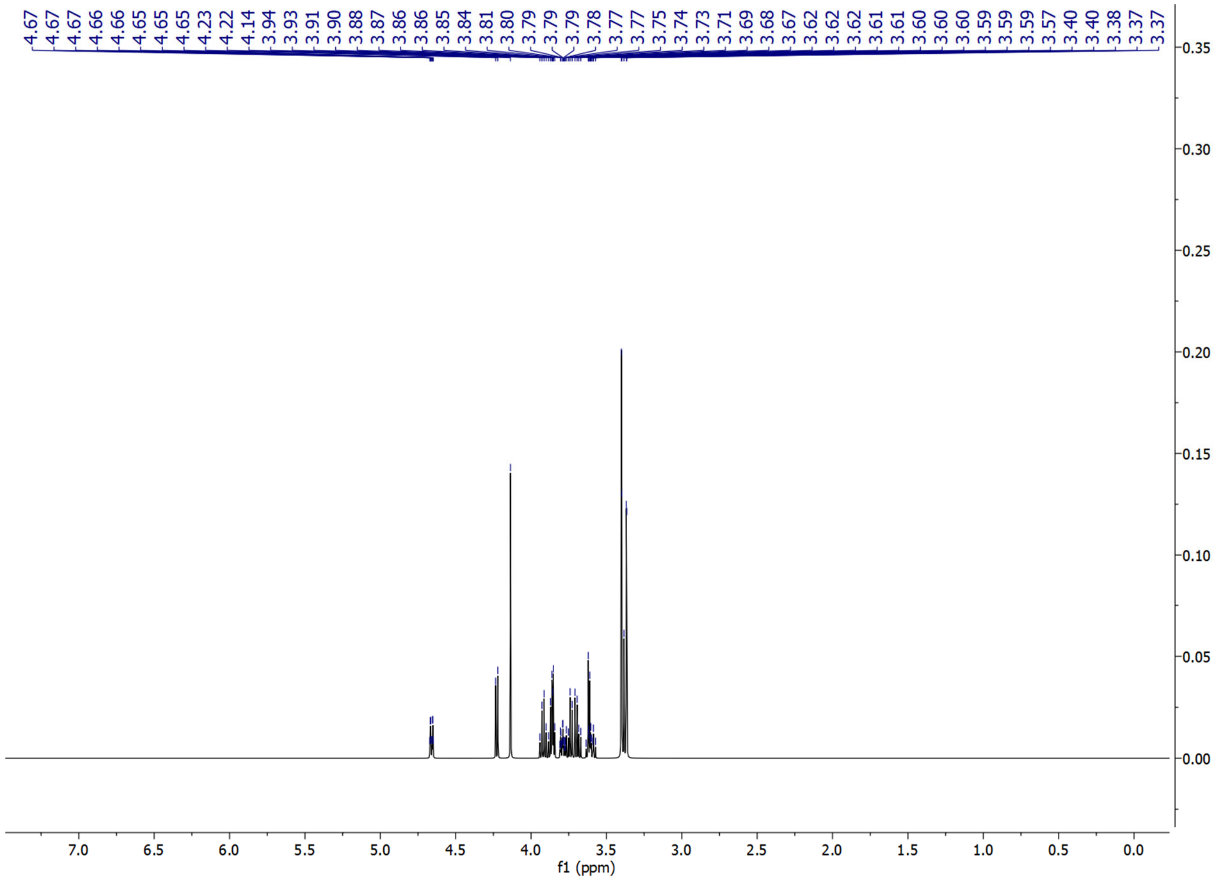

Figure S1. ^{1}H-NMR of CMCS.

^{1}H-NMR of arachidonic acid (AA)

1H NMR, δ 5.52 – 5.41 (m, 6H), 5.41 – 5.30 (m, 2H), 3.64 (qd, 2H), 2.68 (ddp, 6H), 2.26 (t, 2H), 2.10 (dtt, 2H), 2.04 (tdd, 2H), 1.65 (p, 2H), 1.38 – 1.29 (m, 2H), 1.26 – 1.17 (m, 4H), 0.93 – 0.82 (m, 3H)

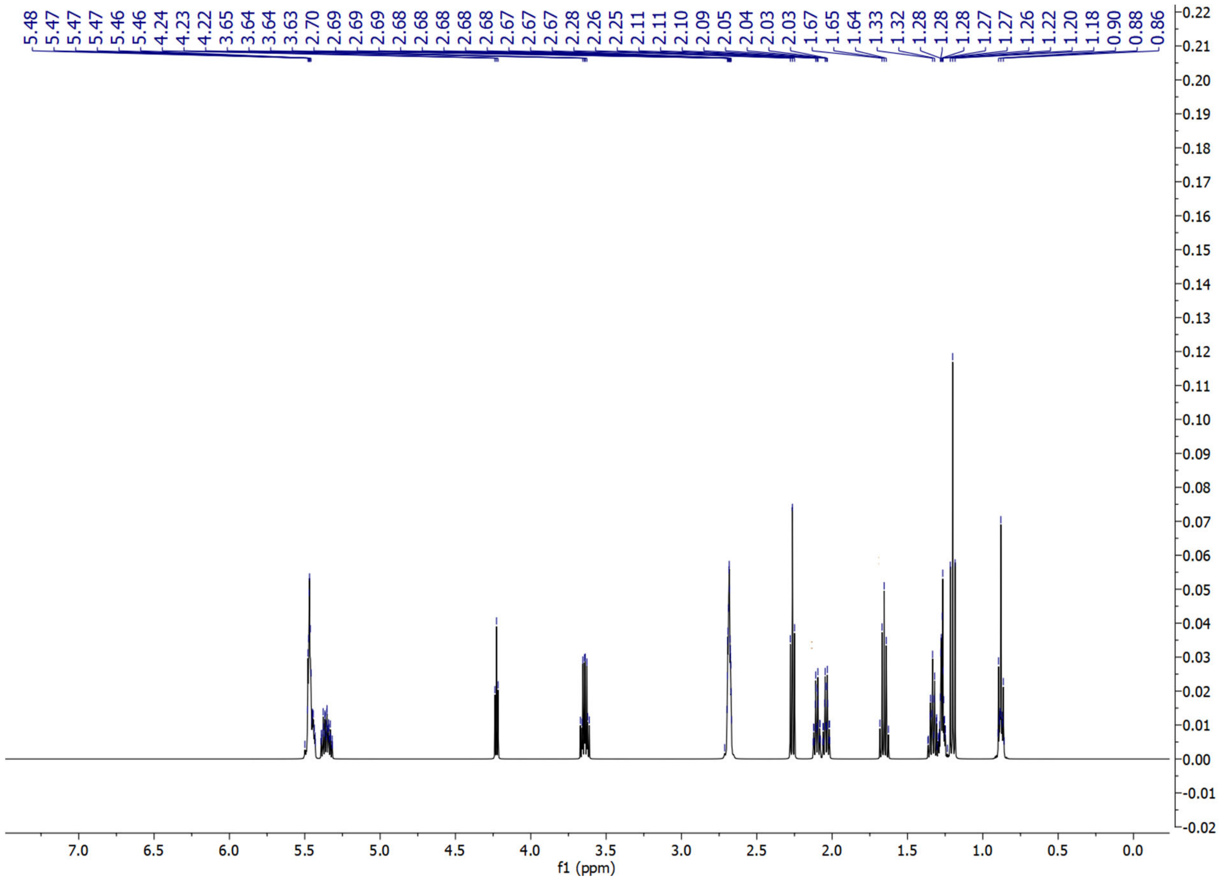

Figure S2. ^{1}H-NMR of AA.

^{1}H-NMR of arachidonic acid-carboxymethyl chitosan (AA-CMCS) conjugate

^{1}H NMR, δ 5.55 – 5.44 (m, 14H), 5.42 – 5.30 (m, 4H), 4.96 (dq, 2H), 4.78 (d, 2H), 4.16 – 4.06 (m, 6H), 3.94 (q, 2H), 3.83 – 3.77 (m, 3H), 3.77 – 3.66 (m, 3H), 3.47 (d, 6H), 3.40 – 3.31 (m, 8H), 2.73 – 2.64 (m, 2H), 2.60 – 2.50 (m, 6H), 2.50 – 2.45 (m, 3H), 2.45 – 2.40 (m, 1H), 2.39 – 2.30 (m, 4H), 2.16 – 2.07 (m, 3H), 2.07 – 1.99 (m, 4H), 1.99 – 1.93 (m, 1H), 1.79 – 1.64 (m, 4H), 1.38 – 1.19 (m, 11H), 0.94 – 0.84 (m, 6H).

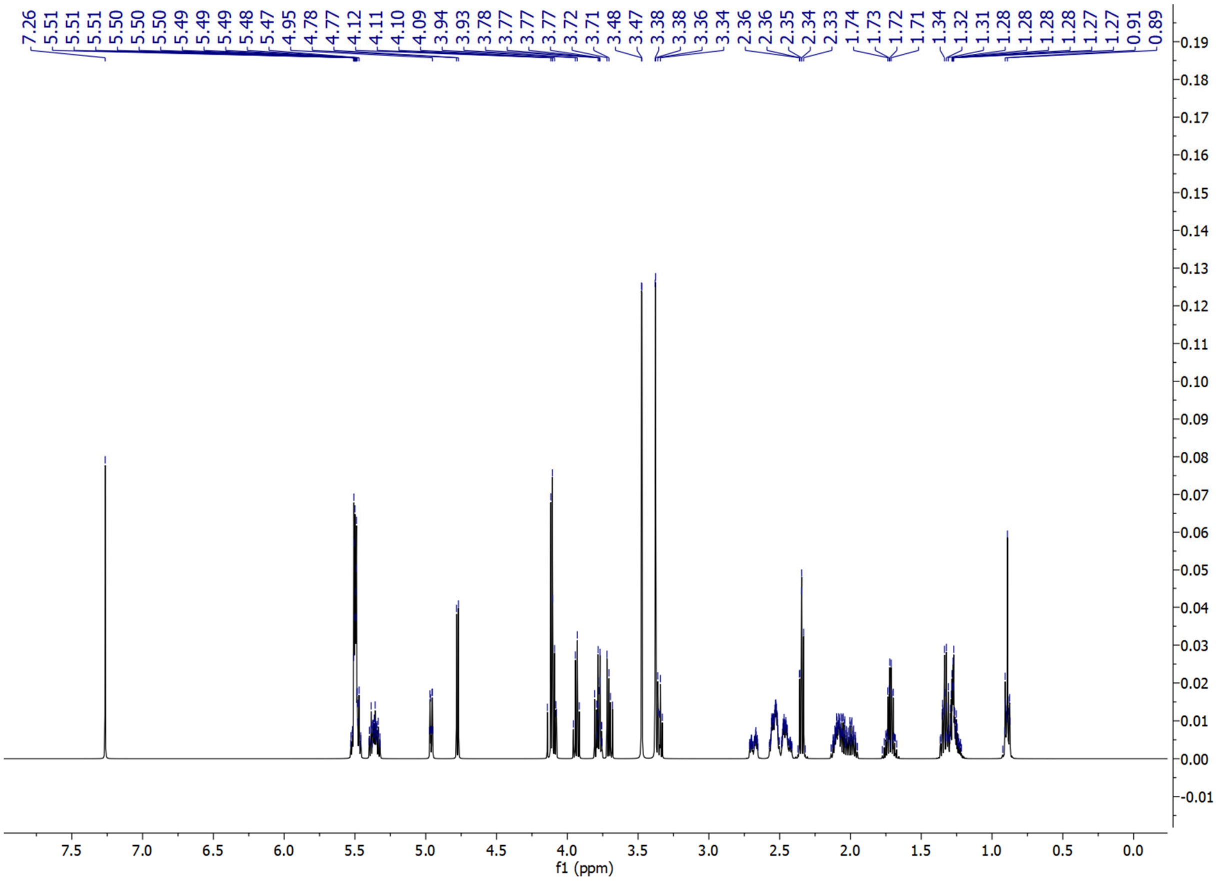

Figure S3. ^{1}H-NMR of AA-CMCS.

2. Experimental Design

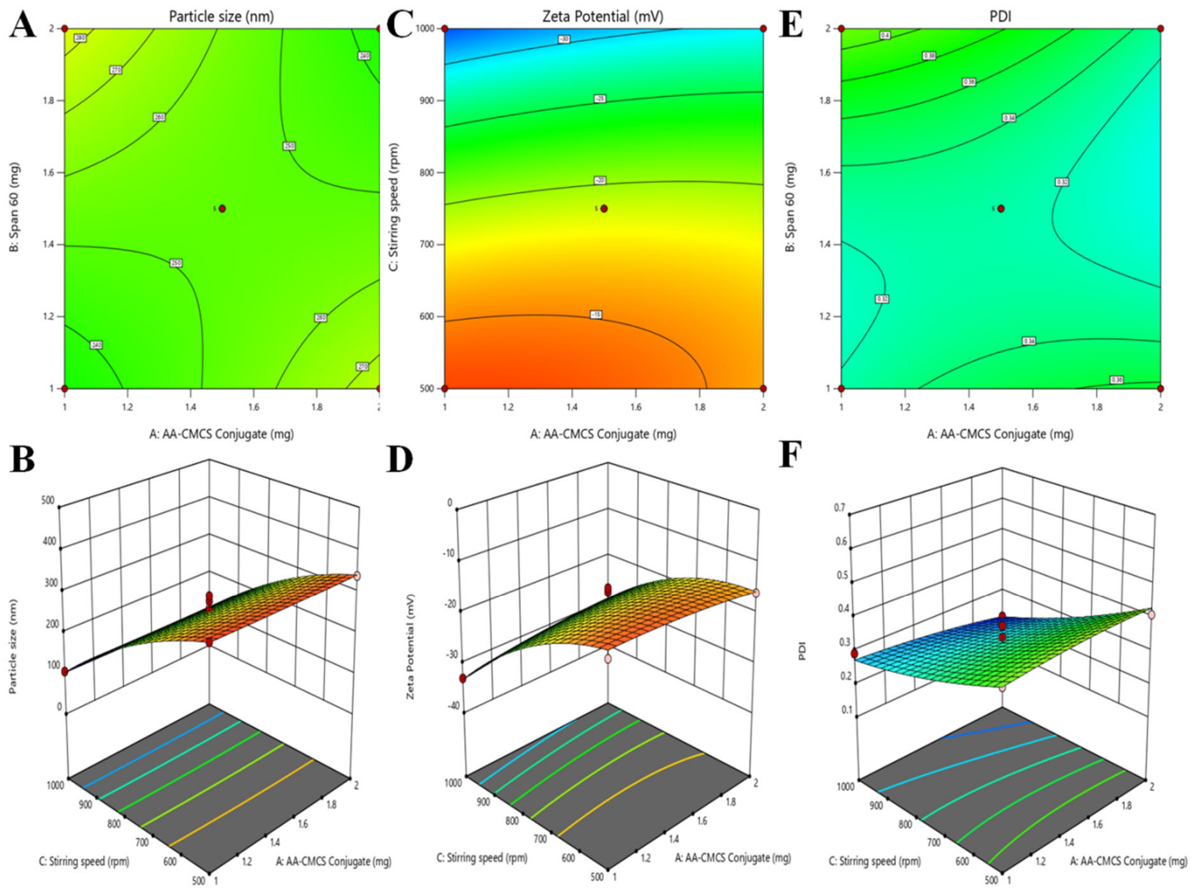

Figure S4. Contour and 3D graphs showing the effect of AA-CMCS conjugate, span 60 and stirring speed on the particle size (A and B), zeta potential (C and D) and PDI (E and F).

3. Entrapment efficiency and drug loading

Table S1. Values of % EE and % drug loaded in novasomes.

Code	% EE	Drug loading (%)
NAC1	59.56±1.453	56.45±2.123
NAC2	67.34±1.089	62.67±2.872
NAC3	71.67±2.981	69.81±1.845
NAC4	56.45±1.652	50.91±1.034
NAC5	78.40±1.789	74.38±.1405
NAC6	81.34±1.021	76.32±1.629
NAC7	49.81±2.098	43.21±2.845
NAC8	90.92±1.567	87.65±2.085
NAC9	82.45±1.043	74.31±2.897
NAC10	80.90±1.498	76.59±3.653
NAC11	67.92±1.619	61.34±1.980
NAC12	75.39±2.923	69.81±2.653
NAC13	63.34±3.678	55.62±4.821
NAC14	53.21±2.937	45.98±2.874
NAC15	59.82±3.452	54.32±2.980
NAC16	78.45±3.765	72.89±3.987
NAC17	80.67±3.591	74.32±3.678

4. Release kinetics of FVM

Table S2. The kinetic parameters of FVM release from novasomes at pH 7.4.

Code	Zero order	First order	Higuchi	Hixon Crowell	Korsmeyer-Peppas	
	R^2	R^2	R^2	R^2	R^2	n
NAC1	0.9815	0.8812	0.9323	0.9924	0.9987	0.415
NAC2	0.9940	0.7760	0.9059	0.9901	0.9989	0.489
NAC3	0.8193	0.7686	0.9452	0.9878	0.9928	0.480
NAC4	0.9863	0.8821	0.9245	0.9933	0.9989	0.430
NAC5	0.9863	0.7821	0.9245	0.9933	0.9989	0.430
NAC6	0.9924	0.8751	0.9113	0.9893	0.9988	0.474
NAC7	0.9858	0.7736	0.9268	0.9886	0.9991	0.425
NAC8	0.9998	0.7726	0.9454	0.9895	0.9989	0.462
NAC9	0.8446	0.7815	0.9635	0.9963	0.9722	0.486
NAC10	0.9780	0.8757	0.9373	0.9883	0.9983	0.490
NAC11	0.9259	0.8810	0.9528	0.9958	0.9605	0.479
NAC12	0.9890	0.7765	0.9181	0.9904	0.9986	0.449
NAC13	0.9987	0.7571	0.8599	0.9752	0.9993	0.444
NAC14	0.9893	0.8776	0.9168	0.9899	0.9980	0.455
NAC15	0.9151	0.8791	0.9531	0.9953	0.9591	0.470
NAC16	0.9872	0.8881	0.9227	0.9966	0.9992	0.435
NAC17	0.9808	0.8926	0.9269	0.9959	0.9966	0.413
